# A Multi-Method Simulation Toolbox to Study Performance and Variability of Nanowire FETs

**DOI:** 10.3390/ma12152391

**Published:** 2019-07-26

**Authors:** Natalia Seoane, Daniel Nagy, Guillermo Indalecio, Gabriel Espiñeira, Karol Kalna, Antonio García-Loureiro

**Affiliations:** 1 Centro Singular de Investigación en Tecnoloxías da Información, University of Santiago de Compostela, 15782 Santiago de Compostela, Spain; 2 Nanoelectronic Devices Computational Group, College of Engineering, Swansea University, Swansea, Wales SA1 8EN, UK

**Keywords:** nanowire field-effect transistors, variability effects, Monte Carlo, Schrödinger based quantum corrections, drift-diffusion

## Abstract

An in-house-built three-dimensional multi-method semi-classical/classical toolbox has been developed to characterise the performance, scalability, and variability of state-of-the-art semiconductor devices. To demonstrate capabilities of the toolbox, a 10 nm gate length Si gate-all-around field-effect transistor is selected as a benchmark device. The device exhibits an off-current ($I_{\mathrm{OFF}}$) of 0.03μA/μm, and an on-current ($I_{\mathrm{ON}}$) of 1770 μA/μm, with the $I_{\mathrm{ON}}/I_{\mathrm{OFF}}$ ratio $6.63\times 10^{4}$, a value 27% larger than that of a 10.7 nm gate length Si FinFET. The device SS is 71 mV/dec, no far from the ideal limit of 60 mV/dec. The threshold voltage standard deviation due to statistical combination of four sources of variability (line- and gate-edge roughness, metal grain granularity, and random dopants) is 55.5 mV, a value noticeably larger than that of the equivalent FinFET (30 mV). Finally, using a fluctuation sensitivity map, we establish which regions of the device are the most sensitive to the line-edge roughness and the metal grain granularity variability effects. The on-current of the device is strongly affected by any line-edge roughness taking place near the source-gate junction or by metal grains localised between the middle of the gate and the proximity of the gate-source junction.

## 1. Introduction

Gate-all-around nanowire field-effect transistors (GAA-NW FETs) are one of the main contenders for future CMOS technologies [1] since they provide a better electrostatic control of the channel when compared to fin field-effect transistors (FinFETs) [2], the current architecture adopted by the semiconductor industry. In addition, nanowire based transistor architectures extend beneficial properties of multi-gate devices required in digital circuits such as quasi-1D current transport, largely confined electrical fields, and immunity of threshold voltage from substrate bias [3].

On the other hand, GAA-NW FETs, like all deeply scaled semiconductor devices, are greatly affected by variability issues [4], related to either the fabrication process or material variations that can limit their performance and reliability [5]. Previous studies have shown that in the sub-threshold region, GAA-NW FETs are less resilient to intrinsic sources of variability than FINFETs [6,7]. In addition, the significant degradation observed in the on-region performance of GAA-NW FETs due to line edge roughness variations could be a critical issue for the scaling of these devices [6].

Nowadays, technology computer-aided design (TCAD) tools play a key role in the advancement of the semiconductor industry [8]. The TCAD tools are able to quickly characterise semiconductor devices, not only fabricated but also foreseen, and allow to investigate the impact of changes in materials, designs or fabrication processes. Currently, three-dimensional (3-D) simulations are necessary to appropriately model devices such as FinFETs or GAA-NW, due to the two-dimensional (2-D) nature of the quantum confinement, which increases the computational cost of a study [9]. There are different approaches that can be used in simulations of state-of-the-art semiconductor devices, ranging from the relatively simple and low computationally demanding drift-diffusion method [10], to extremely complex quantum mechanical approaches, such as pseudopotential-based electron quantum transport [11] or the non-equilibrium Green’s functions (NEGF) [12] formalism, that can also be coupled to empirical tight-binding models [13]. The use of fully quantum simulators is computationally prohibitive for statistical studies, being essential a trade-off between the simulation’s accuracy and the calculation time.

In this work, an in-house built finite-element multi-method semi-classical/classical simulation toolbox acronymed VENDES (Variability Enabled Nanometric DEvice Simulator) is used to characterise nano-scaled semiconductor devices including their operational performance and variability. To demonstrate capabilities of the VENDES, the performance and variability of a 10 nm gate length Si GAA-NW FET scaled down from an experimental device [14] is studied and assessed. The paper is organized as follows: the simulation techniques available in VENDES used in this study are described in Section 2. Section 3 analyses the performance and resilience to variability of the 10 nm gate length Si GAA-NW FET. Finally, Section 4 draws the main conclusions of this work.

## 2. Simulation Framework

**Figure 1:**
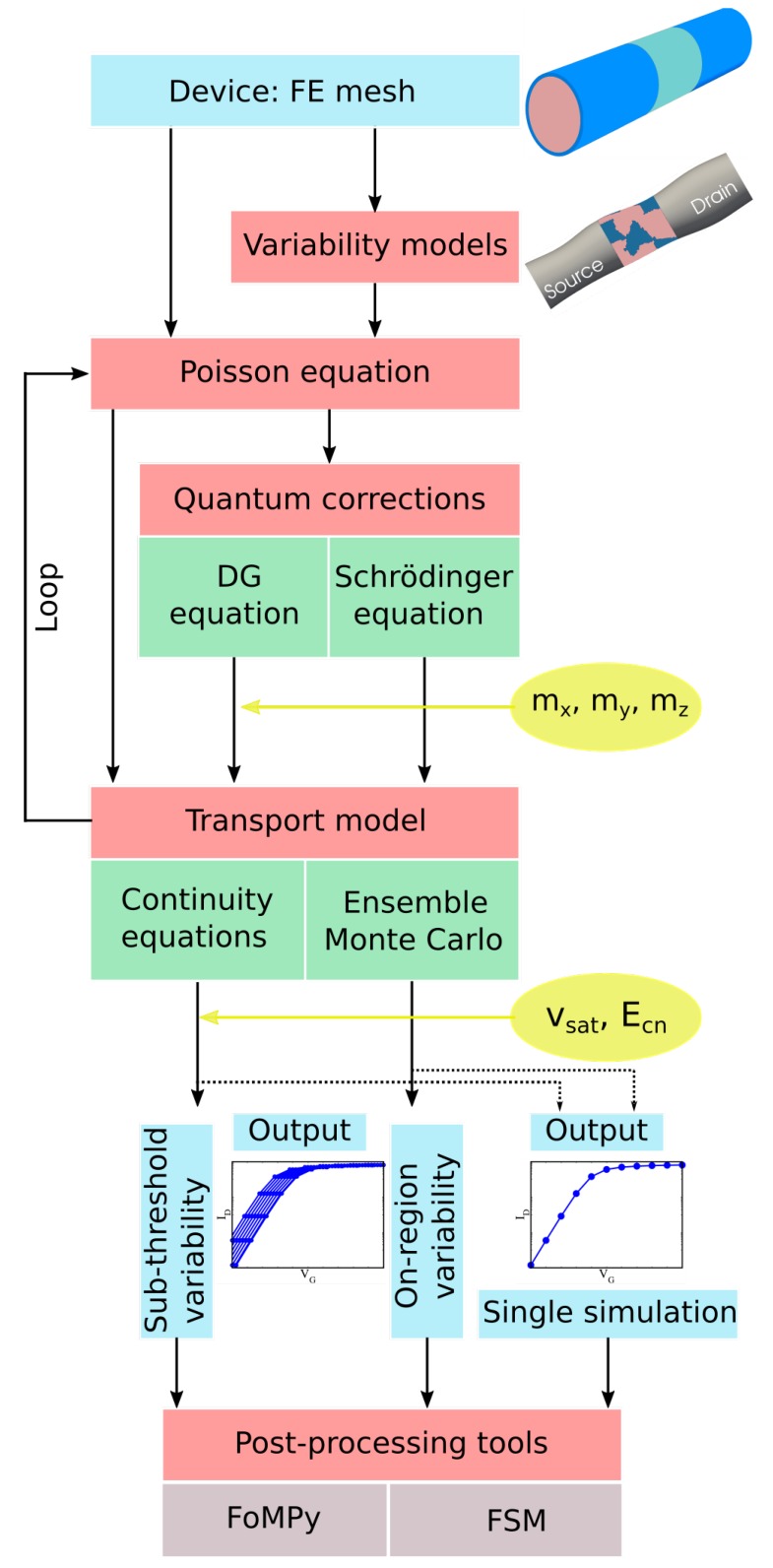
Basic flowchart of VENDES (Variability Enabled Nanometric DEvice Simulator).

VENDES, a 3D finite-element (FE) based device simulator, has been developed jointly at Universidade de Santiago de Compostela (Spain) and at Swansea University (United Kingdom). Figure 1 shows the basic flowchart of the VENDES toolbox.

The starting point is the generation of the FE mesh via the open source software Gmsh [15]. The FE method allows not only an accurate description of complex simulation domains, as in the case of elliptic cross-section shaped GAA-NW FETs [16], but also the possibility of introducing realistic deformations to the device dimensions. This capability of accurate geometrical description is crucial in the modelling of variability effects because a correct distribution of potential and carrier density is essential to predict the experimentally observed behaviour. Note that, for devices deeply scaled into the nanometre regime, the size of device variations and deformations can be comparable to critical device dimensions. The sources of variability included in VENDES either alter the structure dimensions or modify some physical properties affecting the device nodes and are described in detail in Section 3.2.

The classical electrostatic potential, $V_{cl}$, is obtained from the Poisson equation solution on every node of the 3D FE tetrahedral mesh:

(1) $$div\left(\varepsilon \left(r\right)\nabla V_{cl}\left(r\right)\right)=q\left(p\left(r\right)-n\left(r\right)+N_{D}^{+}\left(r\right)-N_{A}^{-}\left(r\right)\right),$$

where r=(x,y,z) is the spatial coordinate, ε(r) is the dielectric constant of the material, n(r) and p(r) are the electron and hole densities and $N_{D}^{+}\left(r\right)$ and $N_{A}^{-}\left(r\right)$ are the effective doping concentrations of donors and acceptors, respectively.

Quantum corrections are incorporated in VENDES via two different techniques: i) the 3D density gradient (DG) equation and ii) the 2D Schrödinger (SCH) equation. In the first case, the DG quantum potential for electrons, $V_{dg}\left(r\right)$ [17], is obtained as:

(2) $$V_{dg}\left(r\right)=2\left[b_{n}\right]\frac{\nabla^{2}\sqrt{n(r)}}{\sqrt{n(r)}}=\phi_{n}\left(r\right)-V_{cl}\left(r\right)+\frac{k_{B}T}{q}\ln\left(\frac{n(r)}{n_{i}\left(r\right)}\right),$$

where

(3) $$\begin{array}{c} \left[b_{n}\right]=\frac{\hbar^{2}}{4qr_{n}}\left(\begin{array}{ccc} 1/m_{x} & 0 & 0\\ 0 & 1/m_{y} & 0\\ 0 & 0 & 1/m_{z} \end{array}\right). \end{array}$$

Here, $\phi_{n}\left(r\right)$ is the quasi-Fermi potential for electrons, $n_{i}\left(r\right)$ is the intrinsic carrier concentration of electron and holes, $k_{B}$ is the Boltzmann constant, *T* is the lattice temperature, *ℏ* is the reduced Planck constant, $r_{n}$ is a dimensionless parameter that models statistical phenomena [18] and $m_{x}$, $m_{y}$ and $m_{z}$ are the DG electron effective masses in the *x*-, *y*- and *z*-directions, respectively [17]. It is important to remark that these effective masses operate as fitting parameters [19] and are not related to the material transport effective masses. The DG effective masses in the transverse directions ($m_{y}$ and $m_{z}$) will account for the strength of the quantum-mechanical confinement of the carriers in the device channel through a threshold voltage shift [20]. The DG effective mass in the transport direction ($m_{x}$) can account for the source-to-drain tunnelling by lowering the barrier of classical electrostatic potential which occurs between the source and the drain when the transistor is operating in the sub-threshold region [20]. The main drawback of the DG based quantum corrections is that they require calibration against either experimental data (when available) or more complex simulation techniques (such as Monte Carlo or Non-Equilibrium Green’s Functions) [21].

On the other hand, the quantum correction method based on the solution of the Schrödinger equation [22] is calibration free. This technique assumes longitudinal and transverse electron effective masses in a minimum of the conduction valley of silicon and accounts for wave-functions penetrating into a surrounding dielectric layer [16,23]. The SCH equation is solved on two-dimensional (2D) slices placed across the device channel using a non-uniform distribution dependent on the gradient of electron density. The 2D quantum-mechanical electron density, $n_{\mathrm{sc}}\left(y,z\right)$, is obtained from the SCH equation eigen-states, $\psi_{i}\left(y,z;E_{i}\right)$, and their corresponding eigen-energies, $E_{i}$, as follows:

(4) $$\begin{array}{c} n_{sc}\left(y,z\right)=g\frac{\sqrt{2\pi m^{*}k_{B}T}}{\pi \hbar }\sum_{i}{\left|\psi_{i}\left(y,z;E_{i}\right)\right|}^{2}\;\exp\left[\frac{E_{F_{n}}-E_{i}}{k_{B}T}\right], \end{array}$$

where $E_{F_{n}}$ is the electron quasi-Fermi level, *g* the degeneracy factor, and $m^{*}$ the electron effective transport mass. The Equation (4) considers Boltzmann statistics and assumes six equivalent valleys for Si (*g* = 6). The SCH quantum correction can be considered ’isotropic’, when the electron effective mass in silicon is taken to be average of longitudinal and transverse electron effective masses, but also ’anisotropic’, when longitudinal and transverse electron effective masses that are dependent on the valley orientation are considered. In that case, Equation (4) is solved separately for each of the three Δ valleys, taking into account the different sub-band edges (i.e., appropriate energy levels) for the different valleys, obtaining a different $n_{\mathrm{sc}}\left(y,z\right)$ for each valley (*g* = 2) and $m^{*}$ will be dependent on the channel orientation which can be 〈100〉 or 〈110〉 as shown in [24].

The electron density, $n_{\mathrm{sc}}\left(y,z\right)$, calculated on the 2D slices, is interpolated to a 3D device density domain to obtain $n_{\mathrm{sc}}\left(r\right)$. The resulting SCH quantum correction potential, $V_{\mathrm{sc}}\left(r\right)$ [22], is as follows:

(5) $$\begin{array}{c} V_{sc}\left(r\right)=\frac{k_{B}T}{q}\log\left(n_{sc}\left(r\right)/n_{i}\left(r\right)\right)-V_{cl}\left(r\right). \end{array}$$

Note that, in the anisotropic SCH quantum correction, a separate $V_{sc}\left(r\right)$ is obtained for each valley.

To simulate the transport inside the channel of the device, VENDES has implemented two different carrier transport methods: i) the drift-diffusion (DD) approach and ii) an ensemble Monte Carlo (MC) technique. The DD approach couples the electrostatic potential obtained from the quantum corrected solution of Poisson equation with the current continuity equation for electrons in order to obtain the electron current density, J${}_{n}\left(r\right)$, as:

(6) $$J_{n}\left(r\right)=-q\mu_{n}\left(r\right)n\left(r\right)\nabla \left(\phi_{n}\left(r\right)\right),$$

(7) $$div(J_{n}\left(r\right))=qR\left(r\right),$$

where $\mu_{n}\left(r\right)$ is the electron mobility and R(r) is the recombination term (set to zero by default). Note that, the DD method only accounts for the local relationship between the velocity and the electric field and it is unable to correctly represent non-equilibrium transport effects [25]. However, some of the non-equilibrium phenomena can be partially mimicked via appropriate mobility models. To model the carrier transport behaviour in GAA-NW FETs, VENDES uses Caughey-Thomas doping dependent low-field electron mobility model [26] coupled with perpendicular and lateral electric field models [27] which better describe carrier transport at large electric fields. When using these mobility models, the main calibration parameters are a low-field carrier mobility, a critical electric field, $E_{\mathrm{cn}}$, and a saturation velocity, $v_{\mathrm{sat}}$.

The limitations of the DD approach can be overcome by using a semi-classical transport model, the MC technique where an ensemble of particles representing carriers evolves through free flights governed by Newton equations and undergoes scattering events with a probability which is determined quantum-mechanically. The best way to initialise the distribution of carriers in the real space, is to use the quantum corrected potential from the solution of the Poisson’s equation, which results in speeding up the simulation. MC uses the analytical non-parabolic anisotropic approximation [28] for the silicon band structure taking into account three valley minima, *X*, *L*, and Γ, using Herring-Vogt transformation to transform ellipsoidal surfaces to spherical ones in order to simplify a calculation of free flights and scattering events. The MC technique considers carrier scattering in a quantum-mechanical way by using typically Fermi Golden Rule [29] to obtain the transition rates. The following electron scattering mechanisms, important for silicon based devices, are included in VENDES: i) electron interactions with intra- and inter-valley acoustic and non-polar optical phonons [28,30], ii) electron interactions with ionised impurities using Ridley’s third body exclusion [31,32] and static screening [29], and iii) electron interaction with interface roughness using Ando’s 2D potential approach [33]. VENDES uses Boltzmann statistics when solving 3D Poisson equation and determining a final state after electron scattering but, the electron scattering with ionised impurities uses Fermi-Dirac statistics to calculate the static screening by a self-consistent calculation of the Fermi energy and the electron temperature from the average electron density and kinetic energy in a whole real space device simulation domain at each scattering event [34]. The inclusion of Fermi-Dirac statistics into electron scattering with ionised impurities turns to be sufficient to correctly simulate injection of carriers into the channel from a heavily doped source/drain when comparing the results from quantum corrected 3D finite element Monte Carlo device simulations with experimentally measured I-V characteristics in nanoscale FinFETs [29] and nanowire FETs [16].

## 3. Performance and Variability of GAA-NW FETs

In this work, VENDES has been applied to study state-of-the-art nanoscale GAA-NW FETs designed for future digital technology node generations [35]. Section 3.1 presents the GAA-NW FET description and main figures of merit. Section 3.2 shows a thorough analysis of the impact that different sources of fluctuations have on this architecture.

### 3.1. Benchmark Device

**Figure 2:**
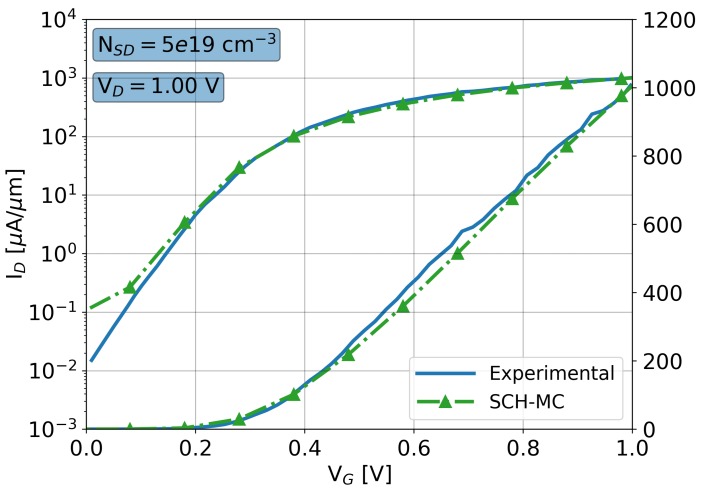
I${}_{D}$-V${}_{G}$ characteristics for a 22 nm gate length GAA-NW FET (gate-all-around nanowire field effect transistor) at a drain bias of 1.0 V comparing experimental results from [14] against Schrödinger equation corrected Monte Carlo (SCH-MC) simulations from VENDES. The maximum source/drain Gaussian doping has been set to $5\times 10^{19}$ cm${}^{-3}$.

The device under study is a 10 nm gate length Si GAA-NW FET with an elliptically shaped cross-section that has been scaled [16] following the ITRS guidelines [35] from an experimental 22 nm gate length device from IBM [14]. Ref. [14] includes TEM images of the fabricated structure and the I${}_{D}$-V${}_{G}$ characteristics that we have used to validate our device. The elliptically shaped cross-section of the transistor body as well as a lateral shape is a result of the advanced fabrication process in which the shape formation is mostly affected by etching. Figure 2 shows a comparison of experimental I${}_{D}$-V${}_{G}$ characteristics of the 22 nm gate length GAA-NW FET versus the simulation results provided by VENDES SCH-MC. The drain bias is 1.0 V. The empirical doping values were not included in [14], so they were reversed engineered following the methodology described in [16]. Note that, the MC device simulations in VENDES are able to accurately reproduce the experimental results in all the active regions of the device, except for at a very low gate bias of 0.0 V, where the MC statistical noise is too high. At the very low gate bias, the DD device simulations are typically used.

**Figure 3:**
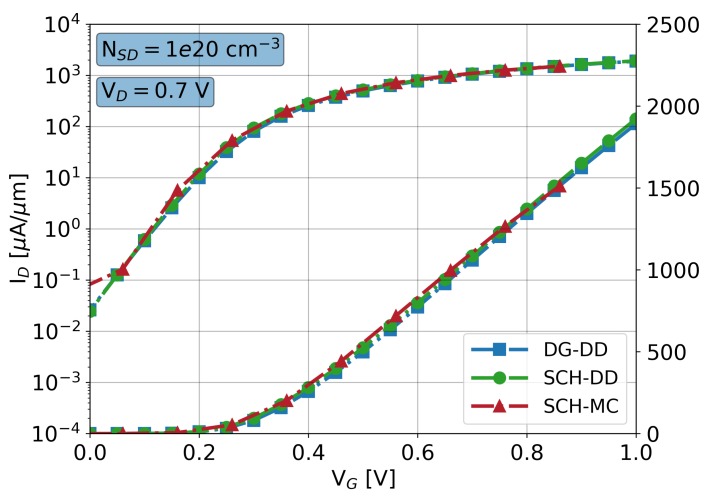
I${}_{D}$-V${}_{G}$ characteristics for a 10 nm gate length GAA-NW FET at a drain bias of 0.7 V comparing simulations results from density-gradient quantum-corrected drift-diffusion simulations (DG-DD), Schrödinger quantum-corrected drift-diffusion simulations (SCH-DD) and SCH-MC. The maximum source/drain Gaussian doping has been set to $10^{20}$ cm${}^{-3}$.

The main dimensions and doping values used to model the 10 nm gate length Si GAA-NW FET are summarised in Table 1. The gate work-function (WF) was set to 4.4 eV. For this device, Figure 3 shows the I${}_{D}$-V${}_{G}$ characteristics at a high drain bias of 0.7 V in both linear and logarithmic scales for DG-DD, SCH-DD and SCH-MC simulations. Note that SCH-MC simulations are calibration free, whereas the DG and DD models need to be properly fitted (see the main calibration parameters in Table 1) in order to achieve the agreement shown in Figure 3. The main figures of merit (FoM) that characterise the I${}_{D}$-V${}_{G}$ characteristics are shown in Table 1.

FoMPy [36,37] is a python-based open source post-processing tool implemented in VENDES (see Figure 1) that automatically extracts the main FoMs of a *I*-*V* characteristics. This tool is very useful when performing statistical studies, where a large ensemble of devices needs to be analysed. In this work, the threshold voltage ($V_{T}$) has been obtained using the second derivation method, the off-current is obtained at a 0.0 V gate voltage, and the on-current has been extracted at a gate bias equal to $V_{T}+V_{\mathrm{DD}}$, being $V_{\mathrm{DD}}$ the supply voltage (set to 0.7 V). The analysed device has a low off-current of 0.03 μA/μm, acceptable for applications in mobile low power devices with a long battery life, and an on-current of 1770 μA/μm, that has been achieved by increasing the maximum S/D doping from $5\times 10^{19}$ cm${}^{-3}$, used in the 22 nm gate length experimental device, to $10^{20}$ cm${}^{-3}$. This increase in the doping has allowed to raise the device on-current by 40% (as previously shown in [38]), at the cost of a slight deterioration in the sub-threshold slope (SS). The device SS is 71 mV/dec, not far from the ideal limit of 60 mV/dec. Therefore, the device doping is one of the key design parameters that needs to be considered when designing a device for a specific application. For transistors aimed at high performance (HP), standard performance (SP) and low power (LP) applications, the $I_{\mathrm{ON}}/I_{\mathrm{OFF}}$ ratio is also a key parameter because it provides a global characterisation of the device operation. The observed $I_{\mathrm{ON}}/I_{\mathrm{OFF}}$ ($6.63\times 10^{4}$) is 27% larger than that of a similar gate length Si FinFET [7] of 10.7 nm.

### 3.2. Variability Models

Several sources of intrinsic device variability are considered in VENDES: i) Metal Grain Granularity (MGG) [39], ii) line edge roughness (LER) [16], iii) gate edge roughness (GER) [7] and iv) random discrete dopants (RD) [40]. These variability sources, together with oxide thickness variations (OTV) and interface trap charges (ITC), were shown to affect FinFETs and GAA-NW FETs the most [5,41].

**Figure 4:**
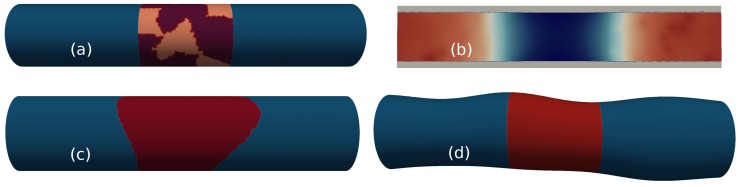
Examples of different sources of variability applied to the 10 nm gate length GAA-NW FET: (**a**) TiN metal profile (with work-function values of 4.4 eV and 4.6 eV) applied to the device gate leading to metal grain granularity (MGG) variations, (**b**) effect of the random dopants (RD) present in the source/drain regions of the device on the device electron concentration, (**c**) device gate affected by gate edge roughness (GER) and (**d**) device body under line edge roughness (LER) variations.

The MGG is modelled by altering the work-function of the device gate so it matches metal grain distributions either observed empirically via KPFM [42], or generated using the Voronoi approach, where the experimental shapes and values of different grain orientations are mimicked [43]. Figure 4a shows an example of a Voronoi TiN metal profile applied to the device gate. The WF values are 4.4 eV and 4.6 eV and their respective probabilities of occurrence 40% and 60%. The average grain size is 5 nm.

RD are introduced in the *n*-type doped S/D regions using a rejection technique from the doping profile (shown in Table 1) of the ideal device. Initially, dopants with their associated charge are distributed on an atomistic grid defined by the location of the atoms. Then, this charge is mapped to the device tetrahedral mesh using the cloud-in-cell technique, in order to generate an atomistic electron density distribution [44], as shown in Figure 4b.

GER and LER are modelled similarly, the device gate (in case of the GER) or the edge of the nanowire (in case of the LER) are deformed according to the shape of a given roughness profile created via the Fourier synthesis method [45]. Two parameters are used to characterise these deformations: i) the root mean square (RMS) height, that sets the amplitude of the roughness, and ii) the correlation length (CL), that accounts for the spatial correlation between the deformations in the different points of the device. Figure 4c,d show examples of devices affected by the GER (with a CL = 11 nm) and the LER (with a CL = 20 nm), respectively. In both cases the RMS height is 0.8 nm.

**Figure 5:**
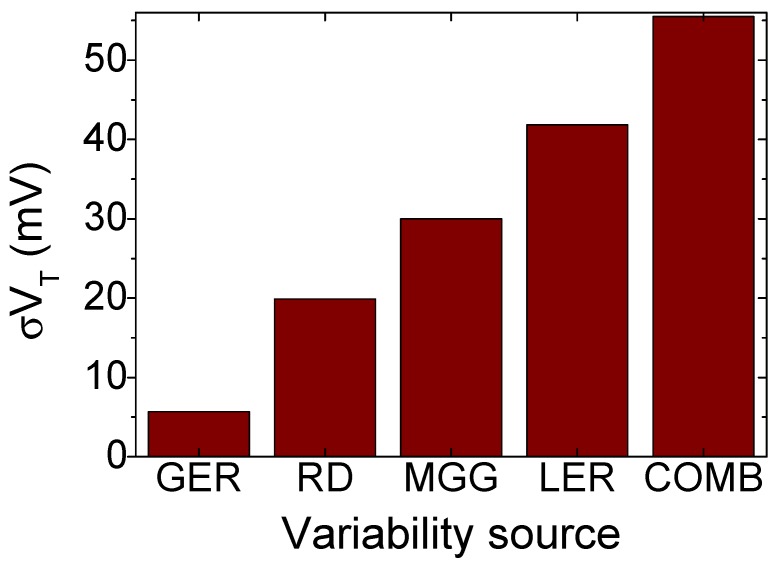
Comparison of the threshold voltage standard deviation due to four different variability sources (GER, RD, MGG and LER) and their combined effect (COMB) in the 10 nm GAA-NW FET.

For each variability source, ensembles of 300 device configurations were created and simulated at a high drain bias of 0.7 V. Figure 5 shows the impact of the aforementioned sources on the 10 nm GAA-NW FET threshold voltage variability. The statistical sum of the four sources of variability (COMB) has also been included as comparison. Results show that GAA-NW FETs are heavily influenced by the LER variability in the sub-threshold region, with σV${}_{T}$ values 1.4 and 2.0 times larger than those of the MGG and the RD, respectively. The GER is the least influential source of variability having its σV${}_{T}$ a 86% lower than that of the LER. Note that the combination of the four sources of variability leads to a V${}_{T}$ standard deviation of 55.5 mV, a value 85% larger than the one observed (σV${}_{T}$ = 30 mV) in a similar gate length Si FinFET [7] of 10.7 nm.

Variability studies are highly computational demanding because they require the simulation of hundreds or thousands of device configurations in order to obtain results with statistical significance. Table 2 shows, for the different simulation methodologies implemented in VENDES, the total times for the solution of one $I_{D}$-$V_{G}$ bias point at a high drain bias of 0.7 V on a single core for two different CPUs. Note that the simulation time for a SCH-MC simulation is around 70 times longer than a quantum-corrected DD study. For that reason, VENDES performs sub-threshold region variability studies (see the flowchart in Figure 1) using either DG or SCH-DD simulations. The reason for this is twofold: i) in the sub-threshold, the electrostatics dominate and quantum-corrected DD simulations are able to provide accurate results and, ii) MC results can be extremely noisy at very low gate biases and lead to incorrect off-current or sub-threshold slope values. However, in the on-region regime, VENDES performs the variability studies via the SCH-MC simulations since the DD approach is unable to capture a non-equilibrium carrier transport even if it is properly calibrated, leading to large over- or under-estimation of the variability [38]. However, it is important to remark (as seen in Figure 3), that both SCH-DD and MC-DD simulations match perfectly at the threshold.

In the quest for the reduction of the computational time, several alternatives have been investigated: i) the parallelisation of the simulation code using a message passing interface (MPI), explained in detail in [46], to take advantage of increasingly available computational infrastructures, such as clusters and supercomputers and, ii) the development of a methodology to predict the impact of the variability sources. A sequential simulation of one $I_{D}$-$V_{G}$ bias point using the SCH-DD method is 960 s, see Table 2 for runs using the Intel(R) Xeon(R) CPU. When using a parallel version of the code with 2 and 4 processors, this time is reduced to 613 s (78 % parallel efficiency) and 363 s (66 % parallel efficiency), respectively.

On the other hand, the Fluctuation Sensitivity Map (FSM) approach [47,48] is a methodology that we developed to predict the impact of the variability sources. This post-processing tool (see the VENDES flowchart in Figure 1) is based on the creation of a map that provides information of the sensitivity of the different regions of a device to a particular source of variability. Once the map is created, it can be used to predict the statistical variability, with a very small error, under different input parameters as shown in Ref. [47]. In a typical variability study, we simulate at least 300 device configurations per variability source and characteristic parameter. This characteristic parameter can be the grain size in the MGG study, or the CL and RMS in a study of LER and GER. For instance, a full MGG variability study will require simulations of several grain sizes (a minimum of three). Therefore, the total computational cost of this full study using the SCH-DD or SCH-MC methods will be 300 h and 31500 h, respectively (for the Intel(R) Xeon(R) CPU in the sequential case). These times can be reduced by 66% using the FSM because once one set of 300 device configurations is simulated and used to create the FSM (which can take up to 2 min), the map can be used to predict the variability results for the remaining grain sizes without any further statistical computations.

**Figure 6:**
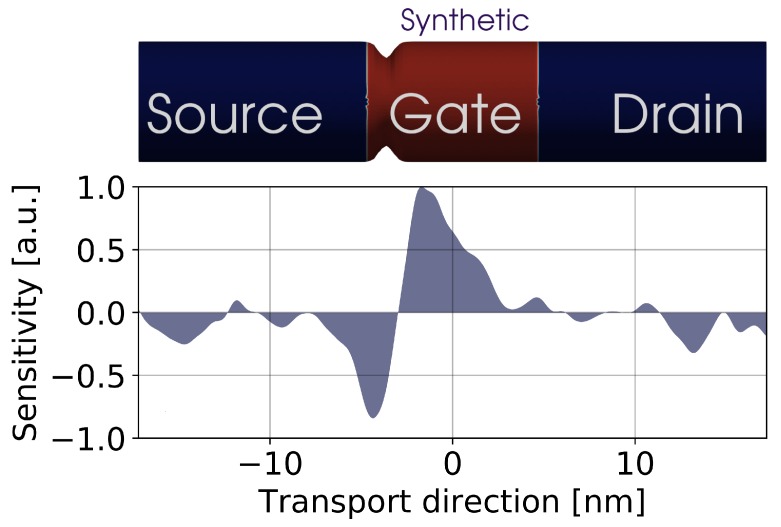
(**Top**) Schematic of a 10 nm gate length GAA-NW FET affected by a localised LER deformation. (**Bottom**) 1D on-current FSM generated from the simulation of 100 localised LER profiles swept along the device channel at a 0.7 V drain bias.

The FSM can be also used as an assistance in the design of variability-resistant device architectures since it pinpoints to parts of the device the manufacturers should concentrate their efforts on. Figure 6 shows an example of the on-current FSM obtained when a single LER deformation is applied to a specific location of the device, narrowing its width. Using this synthetic deformation, it is possible to sweep all the locations along the device, measuring the changes in the FoM that enable us to the spatial sensitivity to LER variations. Note that, in Figure 6 (bottom), a negative (positive) sensitivity indicates an increase (decrease) in the on-current. The effect of the synthetic deformation on the on-current depends on its position along the transistor. Any change in the NW width happening near the source-gate junction will heavily impact the device on-current whereas if the deformation occurs near the source or drain ends, its impact on the on-current will be minimal.

**Figure 7:**
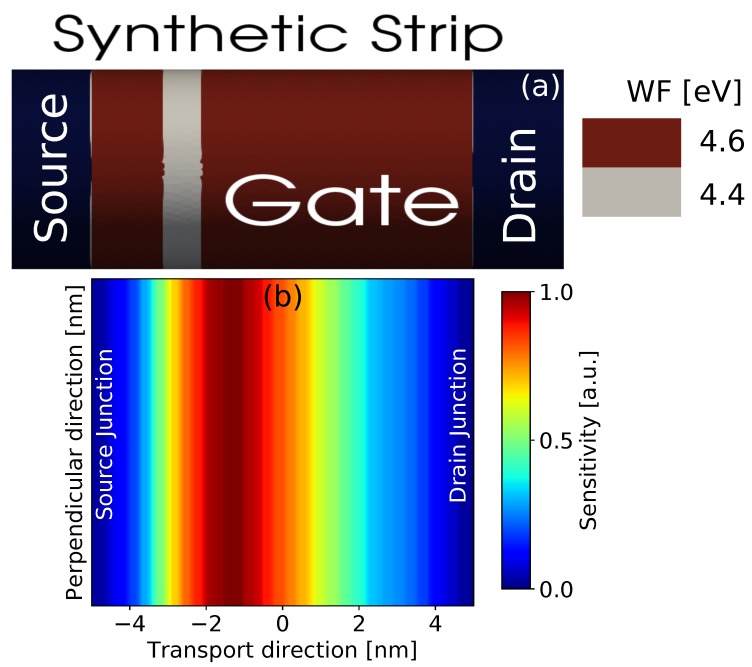
(**a**) Schematic of a 10 nm gate length GAA-NW FET affected by a synthetic MGG profile. The WF is 4.6 eV in all the gate except from a narrow strip (0.1 nm wide) with a WF 4.4 eV. (**b**) 2D on-current FSM generated from the simulation of 100 synthetic gate profiles swept along the device at a 0.7 V drain bias.

Similarly, the FSM can be applied to other sources of variability, like the MGG, taking into account that the generated fluctuation map will now be two-dimensional (2D), in order to characterise the whole device gate. Figure 7a shows a scheme of the device that has a fixed WF value of 4.6 eV in all the gate except from a narrow strip in which the WF is 4.4 eV. This narrow strip is swept along the gate and for each position, the device configuration was simulated at a high drain bias of 0.7 V and the corresponding on-current was extracted. The resulting 2D on-current FSM due to the MGG is shown in Figure 7b. This map allows us to establish that any variation in the WF located between *X* = 0.0 nm (middle of the gate) and *X* = −2.5 nm, will have the largest impact on the device performance. However, the GAA-NW FET will be practically insensitive to WF variations happening in the proximity of the source (*X* = −5.0 nm) or the drain (*X* = 5.0 nm) junctions.

## 4. Conclusions

VENDES, an in-house-built 3D multi-method device simulator, has been used to characterise the performance and resistance to variability of a 10 nm gate length Si GAA-NW FET scaled down from an experimental transistor [14] following ITRS guidelines [35]. The off-current of the device is 0.03 μA/μm, and the on-current of 1770 μA/μm, delivering the $I_{\mathrm{ON}}/I_{\mathrm{OFF}}$ ratio of $6.63\times 10^{4}$. The device SS is 71 mV/dec, not far from the ideal limit of 60 mV/dec. σV${}_{T}$ due to the statistical combination of LER, GER, MGG and RD is 55.5 mV, a value significantly larger than that of a similar gate length Si FinFET of 10.7 nm (30 mV). This larger threshold voltage variability indicates that the variability effects may be another limiting factor for the adoption of the GAA-NW FETs in the future technological nodes.

Finally, the FSM allowed us to determine which regions of the device are the most sensitive to the LER and MGG variations and influence the device characteristics the most. In the case of the LER, the changes in the device width occurring near the source-gate junction will heavily impact the device on-current whereas any deformation happening near the source or the drain, will have a negligible influence on the on-current. In the case of the MGG, the most sensitive region of the device is localised between the middle of the gate and the proximity of the gate-source junction. The information provided by the FSM can be very useful as an aid for the creation of fluctuation-resistant device architectures.

## Figures and Tables

**Table 1:** Device dimensions, doping values, main FoMs and calibration parameters for the 10 nm gate length Si GAA-NW FET.

**Dimensions**	Gate length (L${}_{G}$) (nm)	10.0
	Source and drain (S/D) length (L${}_{S}/D$) (nm)	14.0
	Channel width (W${}_{\mathrm{CH}}$) (nm)	5.70
	Channel height (H${}_{\mathrm{CH}}$) (nm)	7.17
	Equivalent oxide thickness (EOT) (nm)	0.80
**Doping values**	S/D *n*-type doping (N${}_{\mathrm{SD}}$) (cm${}^{-3}$)	$10^{20}$
	S/D doping lateral straggle ($\sigma_{x}$)	3.23
	S/D doping lateral peak (x${}_{\max}$) (nm)	7.80
**Figures of merit**	Subthreshold slope (SS) (mV/dec)	71.0
	Threshold voltage ($V_{T}$) (V)	0.250
	Off-current ($I_{\mathrm{OFF}}$) (μA/μm)	0.027
	On-current ($I_{\mathrm{ON}}$) (μA/μm)	1770
	$I_{\mathrm{ON}}/I_{\mathrm{OFF}}$ ratio	$6.63\times 10^{4}$
**Calibration parameters**	Saturation velocity ($v_{\mathrm{sat}}$) (cm/s)	$1.30\times 10^{7}$
	Perpendicular critical electric field ($E_{\mathrm{CN}})\left(V/\mathrm{cm}\right)$	$9.95\times 10^{5}$
	DG electron mass in the transport direction ($m_{x})\left(m_{0}\right)$	0.50
	DG electron masses in the confinement direction ($m_{y,z})\left(m_{0}\right)$	0.10

**Table 2:** Total time for the solution of one gate bias point as a function on the simulation method. Results have been obtained on a single core for two different CPUs: an AMD Opteron 6262HE @ 1.60 GHz and a Intel(R) Xeon(R) E5-2643 v2 @ 3.50 GHz.

Simulation Method	AMD Time (hh:mm)	Intel Time (hh:mm)
DD	00:28	00:08
DG-DD	01:02	00:20
SCH-DD	00:55	00:16
SCH-MC	70:00	35:00

## Abbreviations

The following abbreviations are used in this manuscript:

DD	Drift diffusion
DG	Density gradient
FE	Finite-element
FinFET	Fin field effect transistor
FSM	Fluctuation sensitivity map
GAA-NW FET	Gate-all-around nanowire field effect transistor
GER	Gate edge roughness
KPFM	Kelvin probe force microscopy
LER	Line edge roughness
MC	Monte Carlo
MGG	Metal grain granularity
RD	Random discrete dopants
